# Amenorrhea, Dysmenorrhea and Kindred Disorders Treated with Ergoapiol

**Published:** 1901-01

**Authors:** 


AMENORRHEA, DYSMENORRHEA AND KINDRED DISORDERS TREATED WITH ERGOAPIOL.

DR. W. A. WEIGHTMAN (*Obstetrics*, June, 1900,) says in the treatment of amenorrhea, dysmenorrhea, and kindred disorders, a practical experience of a number of years has convinced me that ergoapiol (Smith) leads all other emmenagogues in points of efficiency, certainty in action, and rapidity of results.

Other remedies used I have found at least to be very uncertain in their action, and a few successive failures not only cause the physician to lose faith in the value of the preparation, but also cause the patient to lose confidence in the physician. In the use of ergoapiol (Smith) I have yet to meet my first failure. The following cases will give an idea of its merits:

Case I.—M. G., age 19; had never menstruated, very anemic, suffered irregularly from severe pains in the abdomen; local examination gave negative results. I prescribed one capsule of ergoapiol (Smith) every four hours, and three days later menstruation began, and has been regular ever since.

Case II.—L.D., female, age 19; primipara; had not menstruated since confinement; a period of ten months; had tried various emmenagogues without results; consulted me April 27th last; was given ergoapiol (Smith) in doses of one capsule three times a day. Menstruation began on the 30th; was absolutely painless and normal in every respect.

Case III.—M. J., age 22, unmarried; had suffered from dysmenorrhea for several years; had been treated by specialists, but received no benefit. Examination disclosed no displacement, stenosis, or structural cause. Patient was given ergoapiol (Smith) four capsules daily, and has since had no return of the pain, and menstruates regularly.

